# Excited State Vibrational Dynamics Reveals a Photocycle That Enhances the Photostability of the TagRFP-T Fluorescent Protein

**DOI:** 10.1021/acs.jpcb.3c07212

**Published:** 2024-01-29

**Authors:** Atsushi Yabushita, Chia-Yun Cheng, Ying Kuan Ko, Takayoshi Kobayashi, Izumi Iwakura, Ralph Jimenez

**Affiliations:** † Department of Electrophysics, National Yang Ming Chiao Tung University, Hsinchu 300, Taiwan; ‡ Research Institute for Engineering, Kanagawa University, Yokohama 2210802, Japan; § Advanced Ultrafast Laser Research Center, The University of Electro-Communications, Chofu 1828585, Japan; ∥ Department of Chemistry, Faculty of Engineering, Kanagawa University, Yokohama 2218686, Japan; ⊥ JILA, National Institute of Standards and Technology and University of Colorado Boulder, Boulder, Colorado 80309, United States; # Department of Chemistry, University of Colorado Boulder, Boulder, Colorado 80309, United States

## Abstract

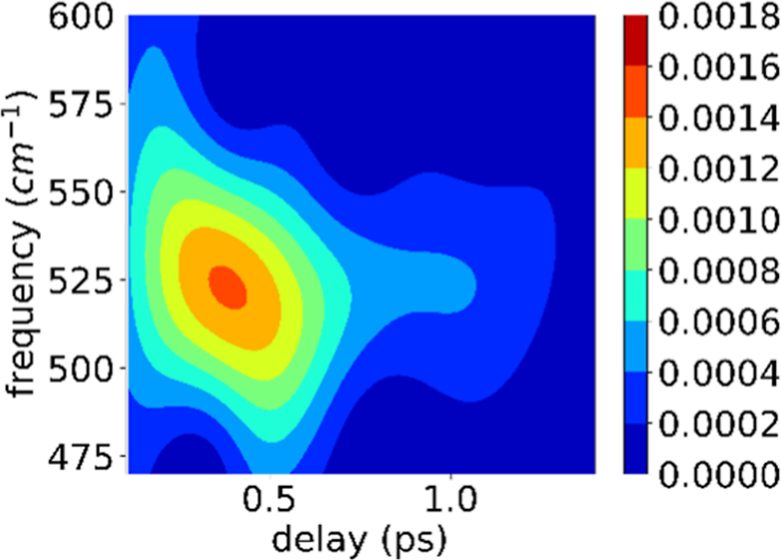

High photostability is a desirable property of fluorescent proteins (FPs) for imaging, yet its molecular basis is poorly understood. We performed ultrafast spectroscopy on TagRFP and its 9-fold more photostable variant TagRFP-T (TagRFP S158T) to characterize their initial photoreactions. We find significant differences in their electronic and vibrational dynamics, including faster excited-state proton transfer and transient changes in the frequency of the *v*_520_ mode in the excited electronic state of TagRFP-T. The frequency of *v*_520_, which is sensitive to chromophore planarity, downshifts within 0.58 ps and recovers within 0.87 ps. This vibrational mode modulates the distance from the chromophore phenoxy to the side chain of residue N143, which we suggest can trigger cis/trans photoisomerization. In TagRFP, the dynamics of *v*_520_ is missing, and this FP therefore lacks an important channel for chromophore isomerization. These dynamics are likely to be a key mechanism differentiating the photostability of the two FPs.

## Introduction

The green fluorescent protein (GFP) discovered in *Aequorea victoria*(1) has enabled the visualization of many biological functions in vivo.^2−7^ The Nobel Prize in Chemistry of 2008 was awarded for the discovery of GFP and its development for tagging proteins in a very wide range of bioscience applications.^8,9^ Subsequent development of GFP-like proteins emitting different colors like red,^10−14^ orange,^15−17^ yellow,^18,19^ and cyan^20−22^ facilitated the simultaneous imaging of multiple processes with multicolor fluorescence microscopy.^23−25^ Red fluorescent proteins (RFPs) are especially useful for imaging in tissues because their red emission is easily separated from cellular autofluorescence, and the red emission scatters less than the shorter wavelength emission of GFP and its yellow variants.

**Figure 1:**
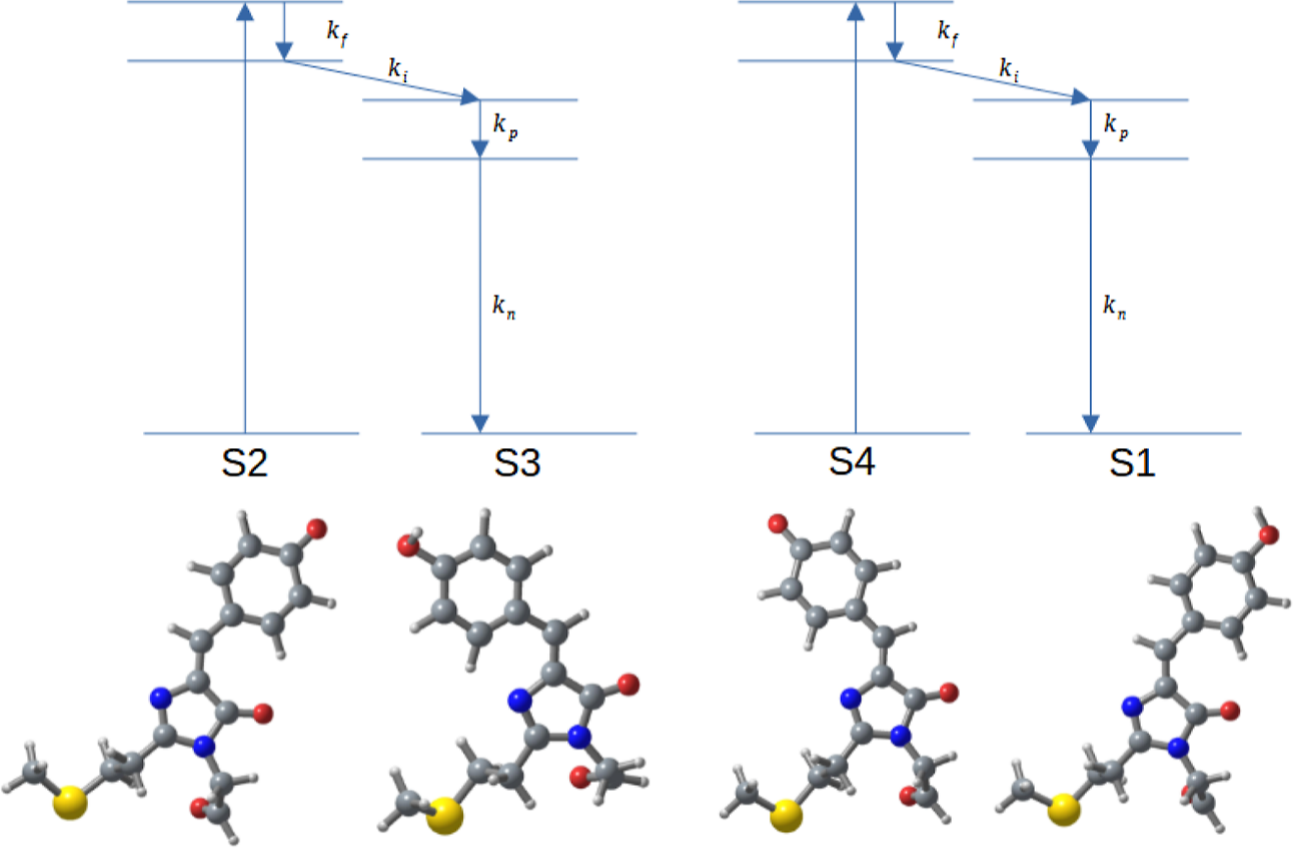
Schematic figure of the energy diagram of TagRFP-T to be observed in the present TA spectroscopy. *k*_f_ (∼100 fs): intramolecular vibrational energy redistribution, *k*_i_ (∼1 ps): photoisomerization, *k*_p_ (∼100 ps): back proton transfer, and *k*_n_ (∼2 ns): emission.

Chudakov and co-workers reported the development of a monomeric RFP, TagRFP, with high brightness, complete chromophore maturation, prolonged fluorescence lifetime, and high pH-stability.^26^ Although the brightness of TagRFP is high, its poor photostability limits applications. The Tsien group used a photobleaching assay on bacterial colonies to screen a small site-directed mutagenesis library of TagRFP and discovered the S158T mutation improved its photostability 9-fold. The new variant is designated TagRFP-T.^27^ Liu et al. performed a crystallographic study of TagRFP-T and proposed a model to explain its superior photostability.^28^ TagRFP-T is thought to consist of equal populations of both trans and cis conformers, both of which can fluoresce when deprotonated. Photoirradiation of TagRFP-T circulates its bright and dim states within a “restoration cycle” among four states. In the photocycle of TagRFP-T, the protonated trans conformer (S1) is a dim state, which can be activated by photoirradiation, converting it to a deprotonated trans conformer (S2) by proton transfer (PT).^29^ The bright S2 state is transiently photobleached by the photoirradiation to produce a protonated cis conformer (S3), which proceeds with photoisomerization and protonation of the chromophore (back PT). Photoirradiation activates the dim S3 state to a deprotonated cis conformer (S4) via PT. The bright S4 state is photobleached by photoirradiation to the dim S1 state via photoisomerization and back PT. Figure 1 shows the optimized structure of the electronic ground state for each state calculated using the Gaussian 16 software package,^30^ with the B3LYP/6-31+g(d) method and basis set.^31,32^ Initial structures for these calculations were taken from the X-ray crystal structure (PDB ID: 3M22). TagRFP-T can reactivate its dim states under photoirradiation, thus leading to improved photostability. In contrast, the chromophore of TagRFP consists only of the trans conformer and is therefore missing a pathway to reactivate its dim state by photoirradiation, thus resulting in photobleaching. This proposed mechanism was based on X-ray crystallography, but the dynamics was not investigated. Here, we resolve the differences in the initial photoreactions of TagRFP and TagRFP-T by femtosecond spectroscopy.

We performed transient absorption (TA) spectroscopy on TagRFP and TagRFP-T using 10 fs broadband visible pulses, which enabled us to observe electronic dynamics by analyzing the relaxation rates of the TA traces probed at all probe wavelengths (490–742 nm). The excitation laser pulse irradiates the sample solution at a laser pulse repetition period of 200 μs. Considering that TagRFP-T is circulating in the solution by natural convection, TagRFP-T to be excited by an excitation laser pulse is not excited by previous laser pulses; thus, the photoexcitation of TagRFP-T is thought to start from its bright states (S2 or S4). Figure 1 shows a schematic energy diagram corresponding to the photoexcited dynamics of TagRFP-T investigated here.

Excitation by a 10 fs ultrashort pulse produces a vibrational wave packet that evolves on the excited electronic potential energy surface at the period of molecular vibration and modulates the TA signal at this period. Therefore, a short-time Fourier transform analysis of the TA trace reports on dynamics of the molecular vibrations. Ultrafast electronic and vibrational dynamics are expected to explain the difference in the photostability of the two TagRFP variants.

## Methods

**Figure 2:**
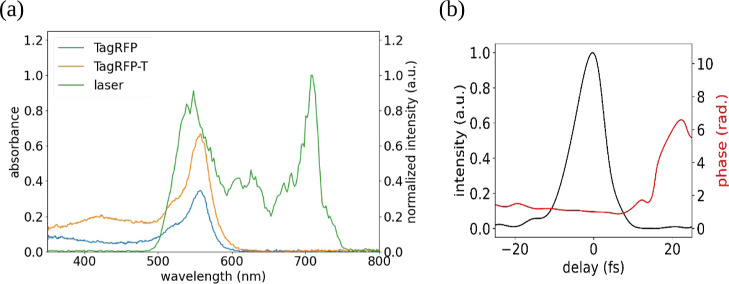
(a) Stationary absorption spectra of the sample solutions of TagRFP (blue) and TagRFP-T (orange) and the spectrum of the 10 fs broadband visible pulse laser (green). (b) Retrieved intensity (black curve) and phase (red curve) of the laser pulse.

We prepared solutions of TagRFP and TagRFP-T in a pH 7.4 PBS buffer. The sample of TagRFP (FP154, Evrogen) was used as received without further purification. The sample of TagRFP-T was prepared with methods described in our previous paper.^31^ Transient absorption spectroscopy was performed in a glass cell (S15-UV-1, GL Science Inc.). Stationary absorption spectra of TagRFP and TagRFP-T are shown along with the spectrum of the 10 fs laser pulse in Figure 2a.

The 10 fs broadband visible laser pulses were generated using our homemade system of a noncollinear optical amplifier (NOPA)^32,33^ (see the Supporting Information for detail). The pulse generated by NOPA was separated into pulses using a beam sampler with a power ratio of 10:1. The pulse with a higher (lower) intensity was used as a pump (probe) pulse in the transient absorption measurement. The optical system was designed to have the same chirp character for the pump pulse and the probe pulse whose spectrum is shown in Figure 2a. The pump and probe pulse durations in the glass cell at the sample position (i.e., after transmission though the input wall of the cell) was adjusted to be as short as 10 fs (see Figure 2b for the retrieved pulse shape). Detail of the pulse characterization are described in the Supporting Information

The delay between the pump pulse and the probe pulse was scanned using an optical delay line (ScanDelay 15, APE Berlin). The probe pulse transmitted through the sample solution was coupled into an optical fiber and then measured by 96-ch lock-in amplifier system. To measure the transient absorption spectrum of the probe pulse, the repetition rate of the pump pulse was decreased by 50% using an optical chopper (MC2000B, Thorlabs Inc.). The detail of the measurement system is described in our previous report.^33,34^

## Results and Discussion

**Figure 3:**
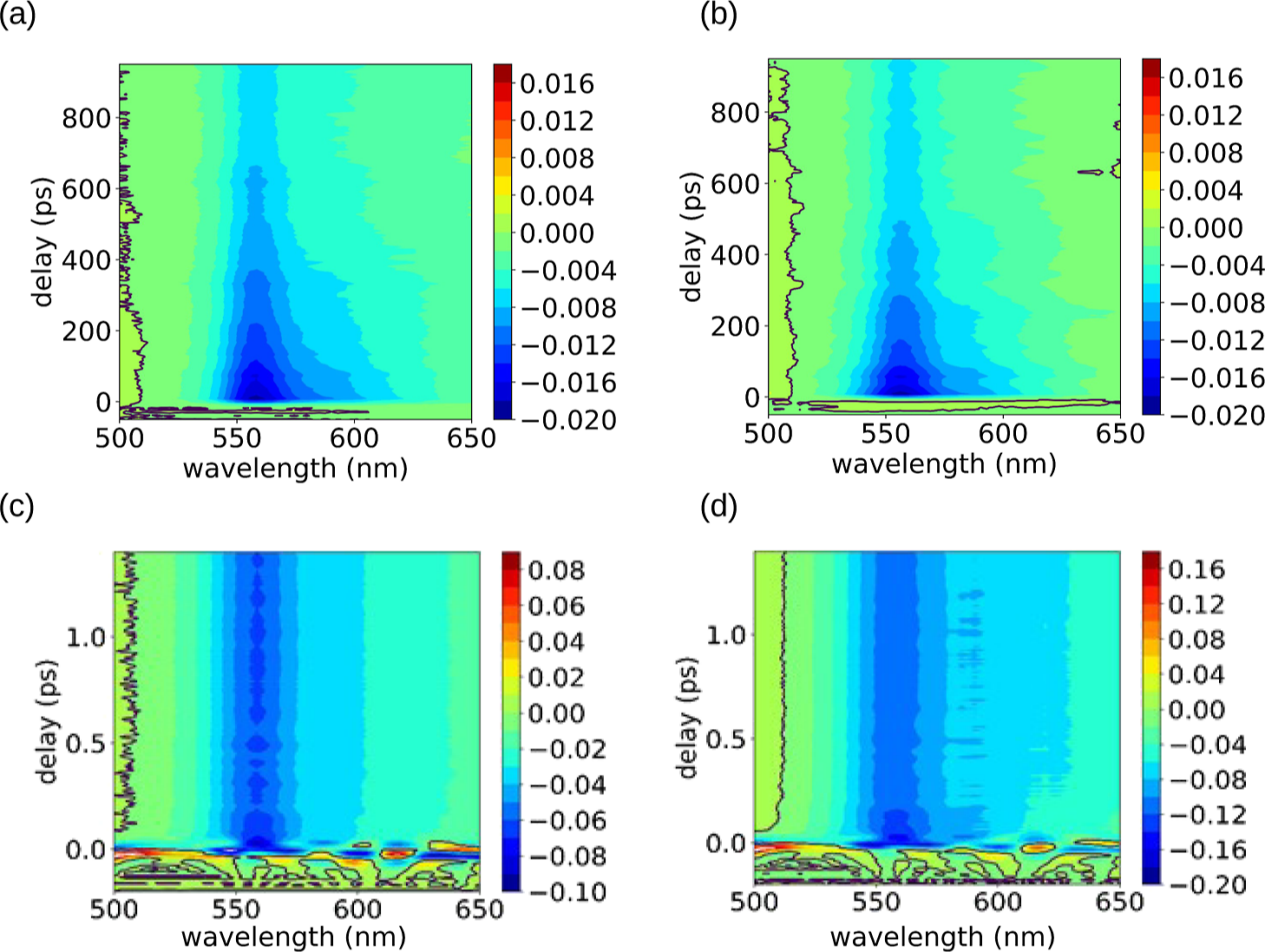
Transient absorption spectra measured in the picosecond region for (a) TagRFP and (b) TagRFP-T. (c) and (d) are those measured in the femtosecond region for TagRFP and TagRFP-T, respectively. Black contours represent the positions where the transient absorption signal is zero.

TA spectra were recorded in the 490–742 nm spectral range with 2.65 nm steps in two time-delay regions. At short times (from −0.322 to 1.393 ps, called the femtosecond region), the delay was scanned with 3.58 fs steps to resolve lifetimes on the 100 fs. At long times (up to 1100 ps, called the picosecond region), the delay was scanned with 0.667 ps steps to resolve lifetimes on the ∼100 ps time scale. Figure 3a,b shows a 2D view of the transient absorption spectrum in the picosecond region for the two samples.

The fluorescence decay rate in the nanosecond region has been reported to be *k*_n_ = 4.3 × 10^–4^ ps^–1^ for both TagRFP^24^ and TagRFP-T.^25^ Global fitting analysis was performed for the TA trace in the picosecond region to estimate the picosecond decay rate *k*_p_. This global fitting analysis was performed using the R software environment with the TIMP library^36^ and a fixed rate of *k*_n_. As an example of the global analysis results performed for the picosecond region, the TA trace probed at 558 nm is plotted with the fitted curve for each sample in the Supporting Information (Figure S4).

For TagRFP and TagRFP-T, *k*_p_ was estimated to be (4.86 ± 0.29) × 10^–3^ ps^–1^ and (5.61 ± 0.58) × 10^–3^ ps^–1^, respectively. This picosecond decay rate of *k*_p_ is assigned to back PT (see Figure 1). The 16% faster decay rate in TagRFP-T indicates that back PT in the cis conformer, which occurs only in TagRFP-T, proceeds ∼30% faster than in the trans conformer, which is present in both FPs. Evolution-associated spectra corresponding to *k*_p_ and *k*_n_ are shown in the Supporting Information (see Figure S2).

By measuring the TA with a few femtosecond resolution, we found that photoreaction of these FPs begins with an ultrafast process in the femtosecond region, which is discussed in more detail below.

Figure 3c,d provides a 2D view of the TA spectra in the femtosecond region for both samples. To estimate the femtosecond decay rate *k*_f_, these TA spectra in the femtosecond region were also analyzed by global analysis, with a fixed rate of *k*_p_ obtained above. For TagRFP and TagRFP-T, *k*_f_ was estimated to be (1.45 ± 0.08) × 10^1^ ps^–1^ and (1.35 ± 0.16) × 10^1^ ps^–1^, respectively. Considering the time scale of 71 ± 13 fs, *k*_f_ is thought to correspond to the intramolecular vibrational energy redistribution (IVR) in the excited electronic state (see Figure 1). Within experimental error, *k*_f_ was found to be the same for both RFPs, indicating that IVR proceeds at a comparable rate for trans and cis chromophore conformations. EAS corresponding to *k*_f_ for each sample is plotted in the Supporting Information (see Figure S3).

**Figure 4:**
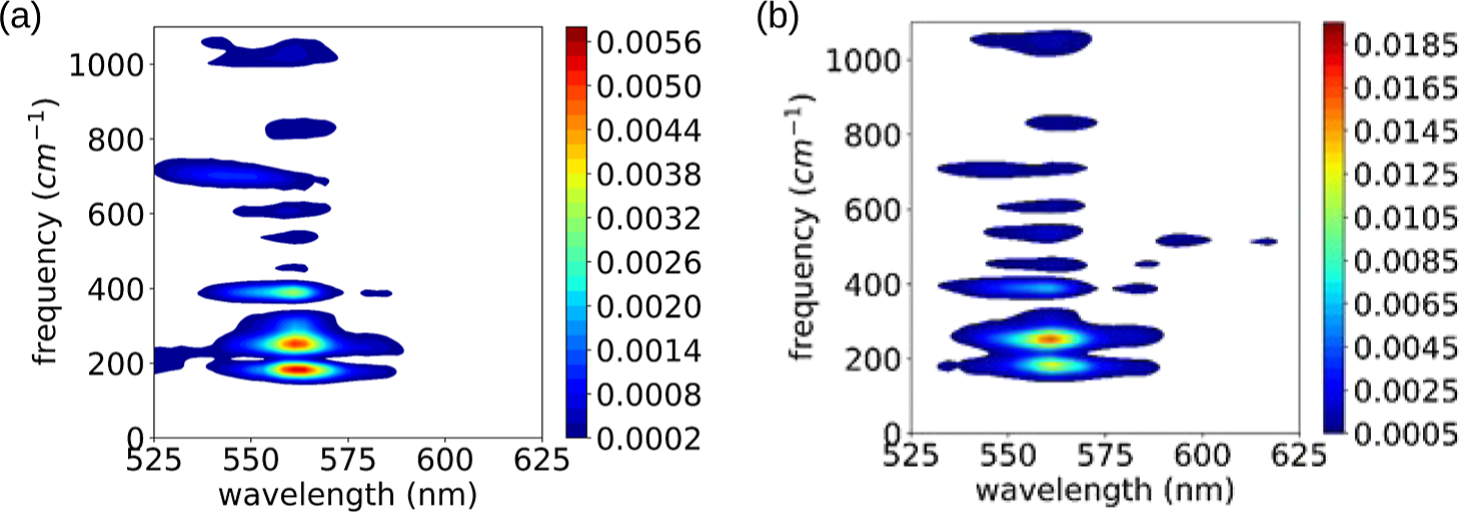
Two-dimensional view of the Fourier power spectra calculated from the TA traces of (a) TagRFP and (b) TagRFP-T.

The TA trace probed at 558 nm is also shown with the fitted curve for each sample in the Supporting Information (see Figure S5). These traces show fine intensity modulations that are reproducible in each measurement scan, which reflects molecular vibrations in the time domain. As seen in the traces, a coherent artifact appears in the region around the zero delay. To avoid noise caused by the coherent artifact, Fourier power spectra of the transient absorption traces were calculated for every probe channel at delays longer than 100 fs (see Figure 4).

At probe wavelengths near 560 nm, an intense signal due to molecular vibrational modes was observed for both samples. In TagRFP-T, a signal around 520 cm^–1^, which we designate *v*_520_, was observed in the probe wavelength region longer than 590 nm, where the stationary absorption spectrum does not extend (see Figures 2 and 4). As discussed before, the transient absorption in this region is dominated by stimulated emission, thus reflecting the dynamics of the electronic excited state. Therefore, the *v*_520_ mode observed here is thought to originate from the electronic excited state. To assign this vibrational mode, we calculated the Raman activity of vibrational modes in the electronic excited state for each of S1–4. The calculation for the first electronic excited state was performed using the Gaussian 16 software,^30^ the TD-B3LYP method, and a basis set of 6-31+G(d). Initial structures for the calculations were taken from the X-ray crystal structure (PDB ID: 3M22). Frequency calculations were performed for all four optimized structures at the same level of theory. All vibrational frequencies were confirmed to be real for the optimized structures. By comparison of the calculated frequency to the measured frequency for the most intense mode observed at ∼1550 cm^–1^ in the Fourier power spectra of the TA trace, the frequency scaling factor was estimated to be 0.982. Calculations were performed without assuming symmetry. 5d functions were used for the d orbital. The Supporting Information shows the normalized Raman activity spectra calculated for S1–4 states of the TagRFP-T chromophore.

**Figure 5:**
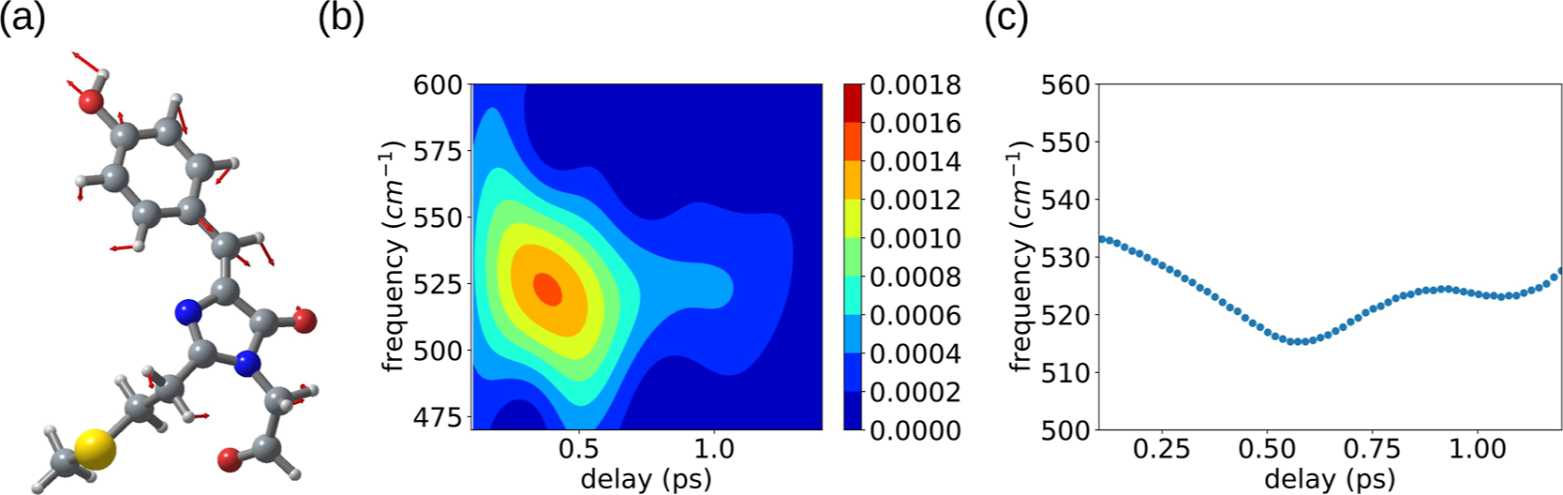
(a) Vibrational motion calculated for the mode of *v*_520_. (b) Calculated spectrogram trace and (c) delay dependence of the *v*_520_ mode frequency calculated by the peak tracking analysis of the calculated spectrogram.

The calculated result shows that *v*_520_ appears only in the S3 state, indicating that excitation of this mode helps to produce the cis conformer of the S3 state. This assignment is consistent with the prediction that the dim S3 state (the protonated cis conformer) only exists in TagRFP-T being produced by photoirradiation of the bright S2 state (the ionized trans conformer). The atomic displacements *v*_520_ are depicted in Figure 5a.

The crystal structure of TagRFP-T shows chromophore interactions with amino acids through its hydroxyphenyl moieties (left top side in Figure 5a). The atomic displacements for the *v*_520_ mode comprise motions that stretch the aromatic ring along directions that modulate the hydrogen bonding interactions with Asn143, Ser/Thr158, and water molecules. We therefore propose that the electronic excitation of TagRFP-T is coupled to vibrational excitation of the *v*_520_ mode, which facilitates protonation and isomerization for the bright S2 state of the trans conformer. The S3 photoproduct state can be subsequently reactivated by photoexcitation. Conversely, for TagRFP, the *v*_520_ mode is not excited, so this FP lacks this channel for promoting photoisomerization; thus, it remains in the trans conformation of the chromophore. As a result, TagRFP is less-efficiently reactivated by photoirradiation than TagRFP-T. Thus, excitation of the *v*_520_ mode in TagRFP-T is key to the difference in photostability between TagRFP and TagRFP-T.

We investigated the dynamics of the *v*_520_ mode by analyzing the transient absorption probed at 596 nm, where the *v*_520_ mode was observed with the highest amplitude (see Figure 4). A spectrogram trace was calculated by the short-time Fourier transform method^35^ using a Blackman window function with full width at half-maximum of 400 fs (see Figure 5b).

Figure 5 shows the delay dependence of the *v*_520_ mode frequency calculated by peak tracking analysis of the calculated spectrogram. The frequency of the *v*_520_ mode shows a downshift in ∼0.6 ps and a recovery in ∼1 ps. The time accuracy of a spectrogram trace is affected by the time-window width of the gate function; thus, we have compared it with numerical simulation data to estimate the time dependency of the vibrational frequency (see the Supporting Information for details). The result shows that the vibrational frequency downshifts to the minimum frequency within 0.58 ps and recovers within 0.87 ps. It is thought to be explained by the following mechanism. Photoexcitation of the S2 bright state triggers photoisomerization and protonation, which proceed with the change of chromophore ligation. The *v*_520_ mode assigned to the vibration of the aromatic ring contains a significant component of stretching along the direction of the H-bonds to these amino acid side chains. Considering that the *v*_520_ mode frequency is recovered after the reaction, the observed frequency shift is thought to be reflecting that the chromophore is twisted by 90° in 0.58 ps and completes the photoisomerization by recovering its planar structure in 0.87 ps. We have performed a calculation of Raman activity for the ground state of the chromophore of TagRFP-T when the chromophore is 90° twisted (see the Supporting Information for details). The calculated result shows that a downshifted mode appears when the chromophore is twisted, which supports this assignment of photoisomerization. This estimated time scale of the photoisomerization is comparable with that in rhodopsin reported by Mathies et al.^36^

## Conclusions

We resolved significant differences in the electronic and vibrational dynamics of the initial photoreactions of TagRFP and TagRFP-T, which we propose are directly related to the mechanism of the nearly order of magnitude difference in their photostabilities. Excited state dynamics on the 100 ps time scale indicate that the proton transfer in the cis conformer of TagRFP-T proceeds ∼30% faster than in the trans conformer. In both FPs, several vibrational frequencies in the ∼200–1000 cm^–1^ region were observed as oscillatory components in the TA spectra. However, the *v*_520_ mode was found only for TagRFP-T in a probe wavelength region, which primarily corresponds to the electronic excited state. Quantum chemistry calculations assign this vibration to a CCC deformation in-plane vibration mode of the phenol ring in the cis conformer. Excitation of the *v*_520_ modulates the distance from the phenol ring moiety to the side chain of residue Asn143, which can then trigger photoisomerization. The delay dependence of the *v*_520_ mode frequency was calculated by a peak tracking analysis of the spectrogram trace. The frequency of the *v*_520_ mode shows a downshift in 0.58 ps, which recovers in 0.87 ps. The frequency shift is explained by the following mechanism. Photoexcitation of the S2 bright state triggers photoisomerization and protonation, which proceed with the change of ligation of amino acid residues to the chromophore. The observed frequency shift reflects chromophore twisting by 90 deg in 0.58 ps and the subsequent photoisomerization recovering planar molecular structure in 0.87 ps. These dynamics can explain why the restoration photocycle only occurs in TagRFP-T. Thus, the analysis with ultrafast spectroscopy has elucidated the reason why the restoration photocycle, which leads to the improved photostability, is available in TagRFP-T but not in TagRFP. This insight into the ultrafast dynamics obtained by the present method may be applicable to efforts to develop FP variants with improved photostability.

## Supporting Information Available

Details of the methods used, evolution associated spectra, transient absorption traces and their fitting curves, normalized Raman activity spectra calculated for S1–4 states of the TagRFP-T chromophore, Raman activity spectra calculated for the 90 degree twisted structure of the TagRFP-T chromophore, and comparison with the numerical simulation result for the peak frequency of the spectrogram trace of measured data (PDF)
