# Supplementary material for: Excited State Vibrational Dynamics Reveals a Photocycle That Enhances the Photostability of the TagRFP-T Fluorescent Protein
Source: J Phys Chem B. 2024 Jan 29;128(5):1188–93. doi: 10.1021/acs.jpcb.3c07212 (PMC10860143; doi:10.1021/acs.jpcb.3c07212)
Supplement: Supplementary file 1 — jp3c07212_si_001.pdf [file jp3c07212_si_001.pdf]

Supporting Information

Excited State Vibrational Dynamics Reveals a Photocycle That Enhances the Photostability of the TagRFP-T Fluorescent Protein

Atsushi Yabushita[1,2*], Chia-Yun Cheng[1], Ying Kuan Ko[1], Takayoshi Kobayashi[1,3], Izumi Iwakura[4], Ralph Jimenez[5,6]

[1]Department of Electrophysics, National Yang Ming Chiao Tung University, Taiwan 300

[2]Research Institute for Engineering, Kanagawa University, Japan 2210802

[3]Advanced Ultrafast Laser Research Center, The University of Electro-Communications, Japan 1828585

[4]Department of Chemistry, Faculty of Engineering, Kanagawa University, Japan 2218686

[5]JILA, National Institute of Standards and Technology and University of Colorado Boulder, USA 80309

[6]Department of Chemistry, University of Colorado Boulder, USA 80309

Email: yabushita@nycu.edu.tw

10 fs broadband visible pulse laser

The 10 fs broadband visible laser pulses were generated as follows. A Ti:sapphire regenerative amplifier (Legend, Coherent Corp.) generates a near infrared (NIR) femtosecond laser pulse (pulse duration 35 fs, center wavelength 800 nm, pulse energy 0.5 mJ, repetition rate 5 kHz). The NIR pulse was separated into two pulses using a beam sampler with a power ratio of 10:1. The pulse with higher intensity was focused into a BBO crystal with thickness of 0.1mm to generate second harmonic to be used as a pump pulse in a non-collinear optical amplifier (NOPA). The pulse with lower intensity was focused into a 1 mm thick sapphire plate for self-phase modulation (SPM) to generate broadband spectrum and the SPM pulse transmitted through a short pass filter (FES0750, Thorlabs Inc.) to be used as a seed pulse in the NOPA. Both pump pulse and the seed pulse were focused into a BBO crystal with thickness of 1mm for NOPA. The amplified seed pulse and the remainder pump pulse were reflected by a concave mirror to amplify the seed pulse again.

The seed pulse amplified twice in the BBO crystal travels through a pulse compressor which consists of a chirped mirror (Custom designed, Tokai Optical Co. Ltd.), a diffraction grating (GR25-0305, Thorlabs Inc.), and a deformable mirror (linear PDM, Flexible Optical B.V.).

After passing through the pulse compressor, the pulse was separated into to pulses using a beam sampler with a power ratio of 10:1. The pulse with higher (lower) intensity was used as a pump (probe) pulse in the transient absorption measurement. The optical system was designed to have the same chirp character for the pump pulse and the probe pulse whose visible broadband spectrum extends from 500 nm to 750 nm.

Pulse characterization of the 10 fs broadband visible pulse

The glass cell storing the sample solutions in the transient absorption spectroscopy has internal optical path length of 1 mm. The wall of the glass cell has a thickness of 1.25 mm whose material chirp is not negligible for the visible broadband laser pulse. One of the glass cells was broken into two pieces to have a piece of the glass wall. The glass wall was inserted in front of the beam sampler which separates the pulse into the pump pulse and the probe pulse. Both of the pump pulse and the probe pulse were focused into a 20 μm thick β-BaB_2O_4 (BBO) crystal on a quartz substrate to generate a sum-frequency signal scanning delay between the pump pulse and the probe pulse for pulse

characterization by using the second harmonic generation frequency resolved optical gating (SHG-FROG) method.

Figure S1(a) shows the measured SHG-FROG trace. The pulse shape retrieved from this trace for the pulse transmitted through the glass wall is thought to be the same as the one at the sample position in the glass cell (i.e. after transmission though the input wall of the cell). The pulse compression system was adjusted to produce this pulse duration as short as 10 fs (see Figure S1(b)).

(a) (b)

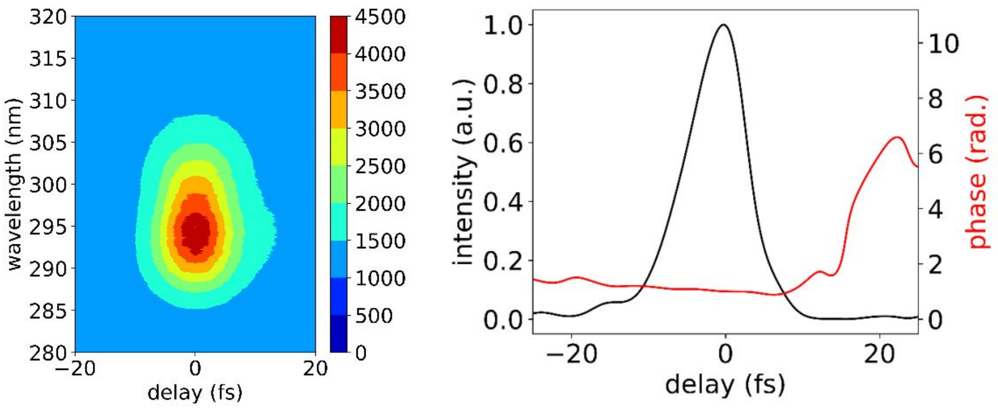

Figure S1 (a) Measured SHG-FROG trace. (b) Retrieved pulse intensity (black curve) and phase (red curve).

Evolution associated spectra

TA spectra (490-742 nm spectral range with 2.65 nm steps) recorded in picosecond region (up to 1100 ps with 0.667 ps steps) were analyzed by global fitting method to estimate lifetime of k_p~200 ps using a reported value of the fixed lifetime of k_n~2 ps. Evolution associated spectra (EAS) corresponding to k_p and k_n are denoted EAS(k_p) and EAS(k_n), respectively, and plotted in

Figure S2.

(a) (b)

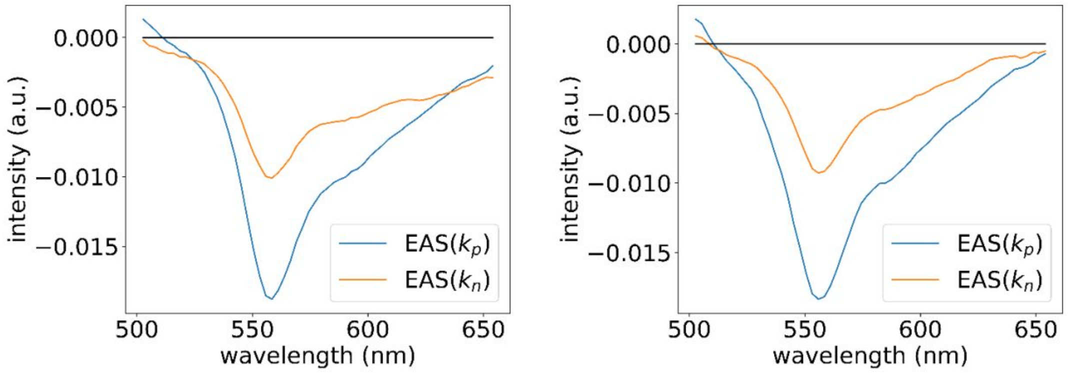

Figure S2 Evolution associated spectra corresponding to the decay rate of k_p (~200 ps) and k_n (~2 ns) for the sample of (a) TagRFP and (b) TagRFP-T.

The TA signal consists of three signal components: induced absorption (IA) of the electronic excited state, stimulated emission (SE) from the electronic excited state, and ground-state bleaching (GB). The former two (IA and SE) reflect the dynamics of the electronic excited state, and the last one (GB) reflects the (recovery) dynamics of the electronic ground state. The first contribution (IA) has positive sign and the latter two (SE and GB) have negative sign in transient absorption measurement. Here, all the calculated EAS have negative sign, which reflects stimulated emission or bleaching. Considering that the stationary

absorption spectra of both samples peak around 560 nm (see Figure 2 (a) of main text), the valley around 560 nm found in both EAS can be assigned to GB. Those absorption bands do not extend beyond 600 nm, therefore the EAS signal at wavelengths longer than 600 nm can be assigned to SE. The TA signal at wavelengths beyond 600 nm reflects the dynamics of the excited state. Spectral shape of EAS is similar between that of k_p and k_n in TagRFP-T. Conversely, TagRFP shows more significant differences in spectral shape between that of k_p and k_n. This observation indicates that, compared with TagRFP, TagRFP-T in the transition corresponding to k_p more closely maintains its original molecular conformation, thus facilitating back PT for the circular restoration.

TA spectra (490-742 nm spectral range with 2.65 nm steps) recorded in femtosecond region (from -0.322 ps to 1.393 ps with 3.58 fs steps) were also analyzed by global fitting method to estimate lifetime of $k_f \sim 100\ \mathrm{fs}$. EAS corresponding to k_f is denoted EAS(k_f) and plotted in Figure S3.

(a) (b)

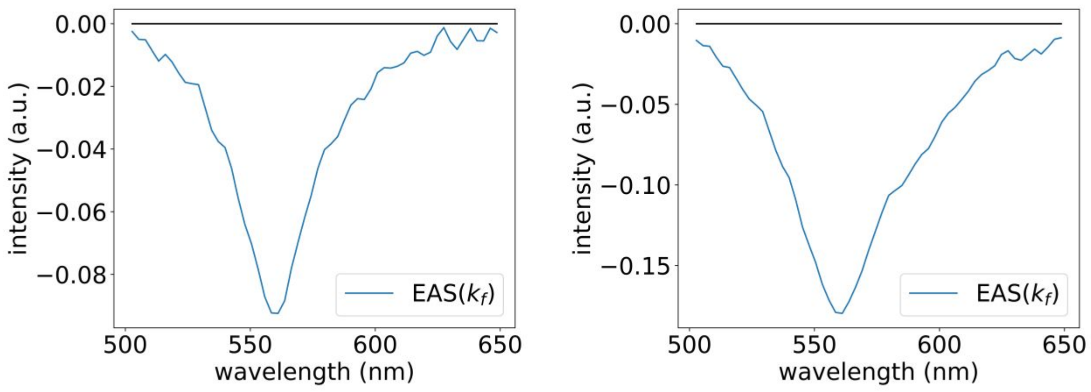

Figure S3 Evolution associated spectra corresponding to the decay rate of k_f (~100 fs) for the sample of (a) TagRFP and (b) TagRFP-T.

The TA spectra of TagRFP-T were found to have a longer tail in the probe wavelength region beyond 560 nm for EAS(k_f). This result implies that photoexcitation of TagRFP-T displaces a wider range of vibrations, which is also consistent with its slightly reduced peak extinction coefficient and slightly wider emission spectrum compared to TagRFP.

Transient absorption traces and their fitting curves

Transient absorption (TA) traces were recorded in the 490-742 nm spectral range with 2.65 nm steps in two time-delay regions; femtosecond region from -0.322 ps to 1.393 ps and picosecond region up to 1100 ps. As an example of the global analysis results performed for the picosecond region, the TA trace probed at 558 nm is shown with the fitted curve for each sample (see Figure S4)

(a) (b)

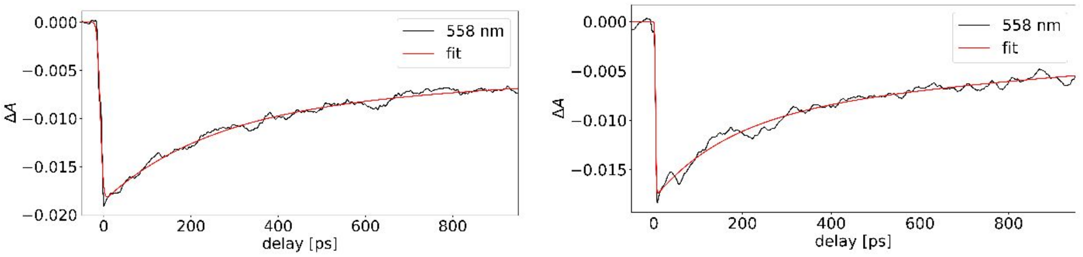

Figure S4 Transient absorption traces probed at 558 nm in the picosecond region are shown with the fitted curve for the sample of (a) TagRFP and (b) TagRFP-T.

As an example of the global analysis results performed for the femtosecond region, the TA trace probed at 558 nm is also shown with the fitted curve for both samples (see Figure S5).

(a) (b)

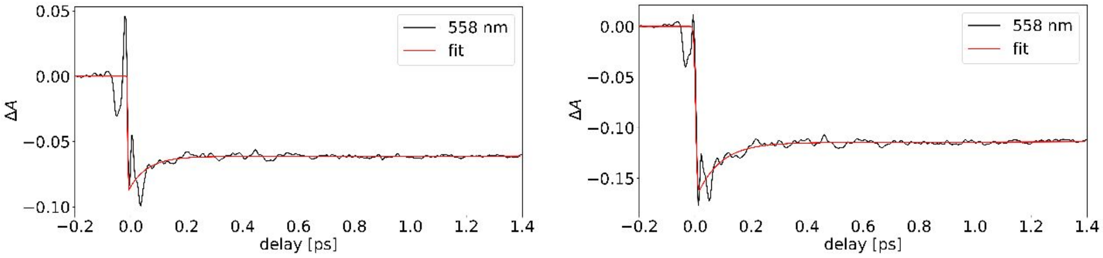

Figure S5 Transient absorption traces probed at 558 nm in the femtosecond region are shown with the fitted curve for (a) TagRFP and (b) TagRFP-T.

Fitting curves calculated in the global analysis were subtracted from the measured TA traces to obtain the high frequency modulation reflecting the molecular vibration in time domain (see Figure S6).

(a) (b)

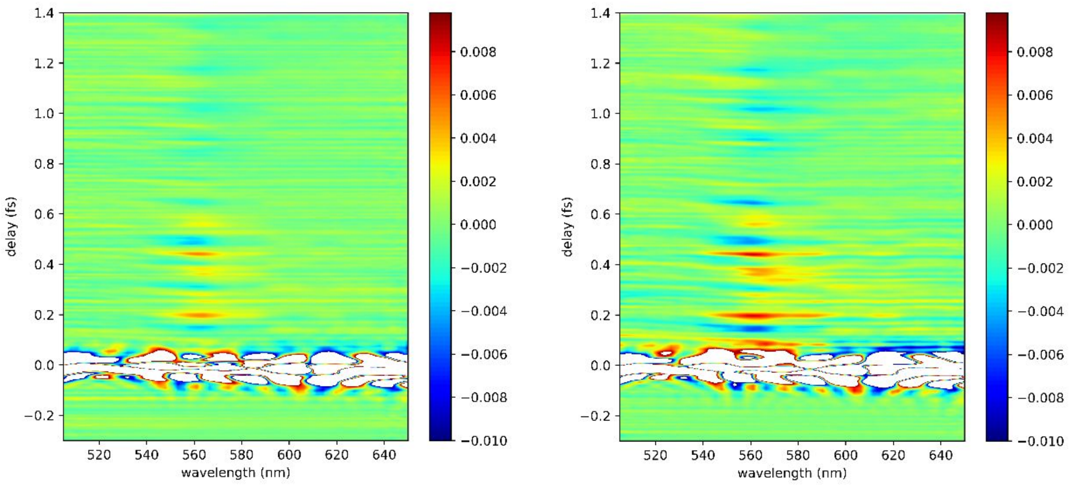

Figure S6 Two dimensional view of the high frequency modulation of the transient absorption traces for (a) TagRFP and (b) TagRFP-T.

Normalized Raman activity spectra calculated for S1-4 states of the TagRFP-T chromophore.

We calculated Raman activity of vibrational modes in the electronic excited state for each of S1-4. The calculation for the first electronic excited state was performed using the Gaussian 16 software, the TD-B3LYP method, and a basis set of 6-31+G(d). Initial structures for the calculations were taken from the x-ray crystal structure (PDB ID: 3M22). Frequency calculations were performed for all four optimized structures at the same level of theory. All vibrational frequencies were confirmed to be real for the optimized structures. By comparing the calculated

frequency to the measured frequency for the most intense mode observed at ~1550 cm^{-1} in the Fourier power spectra of the TA trace, the frequency scaling factor was estimated to be 0.982. Calculations were performed without assuming symmetry. 5d functions were used for the d orbital. Figure S7 shows the normalized Raman activity spectra calculated for S1-4 states of the TagRFP-T chromophore.

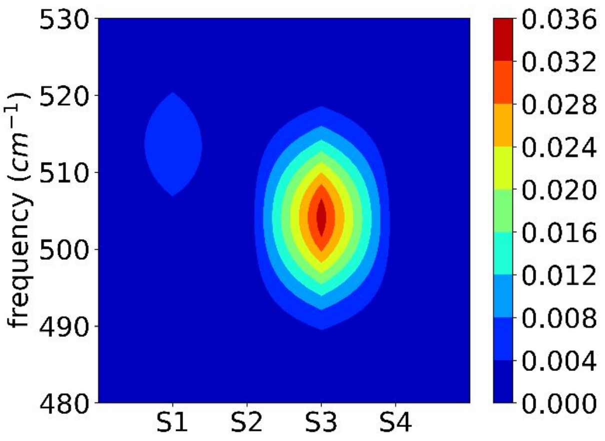

Figure S7 Raman activity in the electronic excited state calculated for S1-S4 states of the chromophore of TagRFP-T.

Raman activity spectra calculated for 90-degree twisted structure of the TagRFP-T chromophore.

We calculated Raman activity of vibrational modes in the electronic ground state for 90-degree twisted structure of the TagRFP-T chromophore. Note that

the calculation for the electronic excited state did not converge till now which is considered to be a future work. The calculation for the electronic ground state was performed using the Gaussian 16 software, the B3LYP method, and a basis set of 6-31+G(d). Frequency calculations were performed for all four optimized structures at the same level of theory. All vibrational frequencies were confirmed to be real for the optimized structures. Calculations were performed without assuming symmetry. 5d functions were used for the d orbital. The calculation result shows that a new mode of ν_{470} with frequency of $470\ cm^{-1}$ becomes Raman active to be comparable with ν_{520} in the twisted structure, which corresponds to hydrogen out of plane mode (see Figure S8). Thus, the observed frequency down shift in the measurement result is thought to be reflecting the molecular structure change to twist the TagRFP-T chromophore.

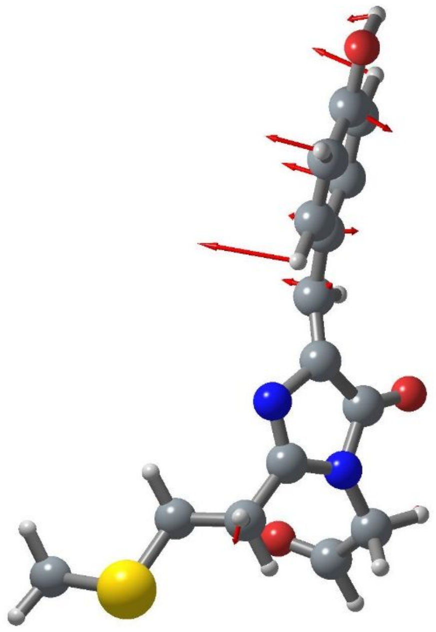

Figure S8 Vibrational motion calculated for the mode of ν_{470}.

Comparison with numerical simulation result for the peak frequency of the spectrogram trace of measured data

The calculated spectrogram of the measured data shows that the frequency of the ν_{520} mode down shifts in ~0.6 ps recovers in ~1 ps. Time accuracy of a spectrogram trace is affected by the time-window width of the gate function, thus we have compared with a numerical simulation data to estimate the time dependency of the vibrational frequency. The time dependent function of vibrational frequency used in the numerical simulation data is as follows,

$$f(t) = \frac{f_{min} + f_{max}}{2} + \frac{f_{max} - f_{min}}{2} cos \frac{2\pi(t - t_0)}{T}.$$

Scanning the parameter of this numerical simulation, time dependence of peak frequency in the measured spectrogram trace was found to be reconstructed by using the parameters of $(f_{min}, f_{max}, t_0, T) = (515\ cm^{-1}, 537\ cm^{-1}, 0.22\ ps, 0.65\ ps)$ for $t < t_0 + T/2$ until when the frequency down shifts to its minimum frequency and $(f_{min}, f_{max}, t_0, T) = (515\ cm^{-1}, 530\ cm^{-1}, 0.22\ ps, 0.65\ ps)$ for $t \geq t_0 + T/2$ after the minimum frequency. Thus, it shows that the vibrational frequency down shifts to the minimum frequency within $t_0 + T/2 = 0.58$ ps and recovers within

$t_0 + T = 0.87$ ps.
